# Dietary intake of monounsaturated and polyunsaturated fatty acids is related to the reduced risk of esophageal squamous cell carcinoma

**DOI:** 10.1186/s12944-022-01624-y

**Published:** 2022-02-27

**Authors:** Yu-Xuan Tang, Wenjing Zhao, Jun Li, Peng Xie, Shuyi Wang, Lubin Yan, Xiangbing Xing, Jiahai Lu, Lap-Ah Tse, Harry Hao-Xiang Wang, Xudong Liu

**Affiliations:** 1 School of Public Health, Guangdong Pharmaceutical University, Address: No. 283 Jianghai Avenue, Haizhu District, Guangzhou, 510310 China; 2 Department of Epidemiology, School of Public Health, Sun Yat-sen University, Guangzhou, China; 3 School of Public Health and Emergency Management, Southern University of Science and Technology, Shenzhen, China; 4 Department of Cancer Prevention and Treatment, Yanting Cancer Hospital, Mianyang, China; 5 Department of General Surgery, Xi’an Aerospace General Hospital, Xi’an, China; 6 Department of Pediatric Surgery, the Sixth Affiliated Hospital, Sun Yat-sen University, Guangzhou, China; 7 Department of Gastroenterology, the First Affiliated Hospital of Sun Yat-sen University, Guangzhou, China; 8 JC School of Public Health and Primary care, the Chinese University of Hong Kong, Hong Kong, SAR China

**Keywords:** Dietary fat, Monounsaturated fatty acid, Polyunsaturated fatty acid, Esophageal squamous cell carcinoma

## Abstract

**Background:**

The relationship of consumption of dietary fat and fatty acids with esophageal squamous cell carcinoma (ESCC) risk remains unclear. This study aimed to explore the relationship of dietary fat and fatty acids intake with ESCC risk.

**Methods:**

This case-control study included 879 incident cases and 892 community-based controls recruited from Southwest China. A food frequency questionnaire was adopted to collect information about dietary information, and intake of fat, saturated fatty acid (SFA), monounsaturated fatty acid (MUFA), polyunsaturated fatty acid (PUFA), and total fatty acid (TFA) was calculated. Odds ratios (ORs) with 95% confidence intervals (95% CIs) were estimated using the logistic regression model.

**Results:**

When comparing the highest with lowest intake quintiles, MUFA (OR: 0.33, 95% CI: 0.21–0.51), PUFA (OR: 0.32, 95% CI: 0.20–0.51), and TFA (OR: 0.44, 95% CI: 0.28–0.70) were related to a reduced risk of ESCC after adjusting for confounders; for non-drinkers rather than drinkers, the intake of SFA was significantly related to a 61% (OR: 0.39, 95% CI: 0.19–0.81) reduced risk of ESCC when comparing the highest with the lowest intake quintiles. Dietary fat was not related to the risk of ESCC.

**Conclusions:**

This study suggested that the more intake of MUFA and PUFA, the lower risk of ESCC, whereas the protective effect of TFA was only observed among non-drinkers. Strategic nutritional programs should consider food rich in unsaturated fatty acids to mitigate the occurrence of ESCC.

**Supplementary Information:**

The online version contains supplementary material available at 10.1186/s12944-022-01624-y.

## Introduction

Esophageal cancer (EC), one of the leading malignant tumors in China and many other countries in the world [1, 2], has received great concern. Over 90% of EC cases in China are esophageal squamous cell carcinoma (ESCC), slightly higher than that of the global average [2]. China, one of the countries with the highest mortality rate of EC [2, 3], showed the five-year survival rate of less than 30% [4]. The high occurrence and lethal nature of EC emphasize the importance of prevention to mitigate the epidemic of EC in a high-risk population.

Esophageal cancer is a multifactorial disease, and the vast majority of the etiologic fraction is attributable to environmental influential factors [5]. Recent research has raised interest in the anticancer properties of nutritional factors such as natural dietary fat and fatty acid in the prevention of ESCC [5, 6]. Natural dietary fat is a triglyceride composed of glycerin and fatty acids [7], and the latter are chemical compounds containing polyunsaturated fatty acid (PUFA), monounsaturated fatty acid (MUFA), and saturated fatty acid (SFA). It is that that fatty acids could promote the aggressiveness of cancer, but on the other side were also identified as potential targets for anticancer metabolism therapies [8]. A systematic review and meta-analysis by synthesizing 15 occident epidemiological studies demonstrated that dietary fat and the specific intake of SFA and PUFA were related to an increased risk of esophageal adenocarcinoma, but there was no significant association with the squamous cell type of esophagus cancer [9]. These findings were supported by the latest prospective cohort among 468,952 USA population after a median follow-up of 15.5 years [10]. However, until now, there has been no relevant study focusing on ESCC from China, which contributed to about 50% of the world’s EC cases.

The dietary habits of the Chinese differ dramatically from those of Euro-Americans in terms of the sources and types of foods and the fatty acid compositions. For example, MUFA is predominantly supplied from foods of animal origin in the western diet [11], whereas MUFA in the Chinese diet is more often obtained from both plant and animal foods [12]. Tao et al. found that fat from plant sources showed a lower risk trend of EC in the Chinese population [13], whereas Hu et al. did not find such association [14]; in addition, neither of them considered the effect of histological types when attempting to disentangle the association [13, 14]. Therefore, the correlativeness between dietary fat and specific fatty acids intake with the risk of ESCC among Chinese is still unclear. Therefore, this community-based case-control study aimed to examine the relationship between intake of dietary fat and fatty acids and ESCC risk among a high-risk population.

## Materials and methods

### Study design and subjects

A community-based case-control study was performed in the Yanting area, one of the high-risk areas of esophageal cancer in China [15–17]. Totally, 942 new incident ESCC cases and 942 controls matched individually with age and sex were successfully recruited between the years 2011 and 2013. Briefly, the incident ESCC cases were recruited consecutively from Yanting Cancer Hospital, which was the designated hospital for upper gastrointestinal tumors prevention and treatment in the Yanting area and one of the cancer-registration sites in China. Eligible cases were local permanent residents aged 40 to 70 years old, had lived there for ≥15 years, and were pathologically confirmed as newly emerged primary incident ESCC. The cases included accounted for about 70% of the total incident ESCC cases in Yanting county during the recruiting period according to the local cancer registry data. For the non-recruited ESCC group, 98.5% were diagnosed according to histopathology. No significant difference in age and gender was found between the ESCC patients included and not included.

The community-based controls were chosen with a multi-stage sampling approach from the same rural communities, and the detailed sampling procedure was reported in previous studies [15–17]. Eligible controls were permanent residents who individually matched to cases in terms of sex and age (within 2 years), underwent upper gastrointestinal endoscopy, had lived in the communities of Yanting county for ≥15 years, didn’t have a history of cancer and mental disorders, and received endoscopic examination within one month to ensure participants not suffering from esophageal cancer and precancerous lesions.

In addition, those whose total calorie intake were out of plus or minus 2 standard deviations from the mean were excluded. Finally, 879 cases (640 males and 258 females) and 892 community controls (621 males and 252 females) were selected in the following analysis. The study got the approval form the Ethical Review Committee for Biomedical Research, School of Public Health, Sun Yat-sen University (No.2019–096). All participating subjects provided written consent following the Declaration of Helsinki.

### Data collection

Questionnaires were employed to obtain the information by means of a face-to-face approach and by trained interviewers with the local dialect. The cases were asked to report the information 5 years before the diagnosis of ESCC and the controls were asked to report the information 5 years before the interview.

A purposely-design questionnaire was adopted to collect information on demographic characteristics (age, gender, education level, marital status, occupation), family cancer history, alcohol drinking, and cigarette smoking. Drinkers were defined as those who had consumed any alcoholic beverage, including beer, wine, and distilled wine, containing at least 20 g of ethanol every week for 6 months or more [18]. Smokers were referred to those who had smoked at least 10 cigarettes or an equivalent amount of tobacco a week for at least 6 months [18]. Those who did not meet the definition of drinkers and smokers were defined as non-drinkers and non-smokers. Height and weight information of the cases 5 years before the diagnosis and the controls 5 years before the interview were collected to measure body mass index (BMI, kg/m^2^).

A validated food frequency questionnaire (FFQ) with 76-item [19] was utilized to collect each food/drink intake from each participant, including intake frequency of each item and intake amount every time. The average daily consumption of each food or drink was estimated by multiplying the intake frequency per day by intake amount every time. The dietary items were then grouped into 21 food groups, and principal component analysis was used to derive dietary patterns. The score of each pattern – the prudent pattern and healthy pattern, was calculated with the weighted approach by using rotated loadings as the weight [20]. The rotated loading of each component is shown in supplementary Table S1. The intake of total calorie, fat, total fatty acid (TFA), SFA, MUFA, and PUFA was calculated according to the Chinese food composition table [21, 22].

### Statistical analysis

Chi-square test and t-test were adopted to examine the differences of categorical or continuous variables between the cases and the controls. The continuous variables of dietary fat and fatty acids intake were then transformed into a categorical variable by quintiles. Odds ratios (ORs) with 95% confidence intervals (CIs) were calculated by using logistic regression before and after adjustment for confounders. The confounders included age (years), gender, body mass index (kg/m^2^), family cancer history, cigarette smoking, alcohol drinking, education, marital status, and total calorie intake (MJ); prudent pattern score (continuous variable) and healthy pattern score (continuous variable) were further adjusted when considering the overall effects of diet. The Wald statistic was calculated to examine the linear exposure-response trend, by putting the median intake of each quintile as a continuous variable into the logistic regression model.

Sensitivity analysis was conducted by considering of energy-adjusted fat or fatty acids intake. The energy-adjusted dietary intake was equal to the residual of the regression of a food component intake on calorie intake plus the mean of a specific food component [23]. Stratified analyses were implemented by cigarette smoking and alcohol drinking. Multiplicative interactions were examined using the likelihood ratio test, with a comparison of the likelihood scores of the two models with and without the interaction terms. All analyses were carried out by using R software (version 3.6.1), with a *p*-value less than 0.05 being statistically significant.

## Results

The characteristics of the cases and the controls are displayed in Table 1. More controls than cases were non-smokers, non-drinkers and without family cancer history (all *P* <  0.05). Average values of BMI and healthy pattern score were all higher in the controls than those in the cases (all *P* <  0.05), whereas average values of total calorie intake and prudent pattern score were greater in the cases than in the controls (all *P* <  0.05). No significant difference in age, gender, and education was observed between the cases and the controls.

**Table 1:** General characteristics of subjects

Characteristic	Controls(*N* = 892)	Cases(*N* = 879)	*P* value
Age, year, mean (S.D.)	60.28 (6.82)	60.14 (6.87)	0.675^*^
Body mass index, kg/m^2^, mean (S.D.)	22.72 (2.49)	21.50 (2.92)	< 0.001^*^
Total calorie intake (MJ), mean (S.D.)	13.34 (3.13)	13.70 (3.76)	0.030^*^
Prudent pattern score, means (S.D.)	167.58 (79.36)	211.31 (118.09)	< 0.001^*^
Healthy pattern score, means (S.D.)	109.47 (85.56)	45.62 (110.01)	< 0.001^*^
Sex, N (%)			0.646^†^
Male	640 (71.75)	621 (70.65)	
Female	252 (28.25)	258 (29.35)	
Body mass index, N (%)			< 0.001^†^
< 18.5 kg/m^2^	34 (3.81)	125 (14.22)	
≥ 18.5 ~ < 23.0 kg/m^2^	455 (51.01)	508 (57.79)	
≥ 23.0 ~ < 25.0 kg/m^2^	258 (28.92)	136 (15.47)	
≥ 25 kg/m^2^	145 (16.26)	110 (12.51)	
Family cancer history, N (%)			0.006^†^
No	715 (80.16)	656 (74.63)	
Yes	177 (19.84)	223 (25.37)	
Cigarette smoking, N (%)			< 0.001^†^
Non-smoker	534 (59.87)	343 (40.13)	
Smoker	358 (40.13)	536 (60.97)	
Alcohol drinking, N (%)			< 0.001^†^
Non-drinker	476 (53.36)	316 (35.95)	
Drinker	416 (46.64)	563 (64.05)	
Education, N (%)			0.137^†^
Lower than primary school	196 (21.97)	183 (20.82)	
Primary school	483 (54.15)	515 (58.59)	
Middle school or above	213 (23.88)	181 (20.59)	
Marital status, N (%)			0.003^†^
Married	805 (90.25)	827 (94.08)	
Others^‡^	87 (9.75)	52 (5.92)	
Dietary intake, mean (S.D.)			
Dietary fat	61.77 (22.11)	65.74 (27.98)	0.001^*^
Total fatty acid	58.21 (20.83)	61.74 (26.23)	0.002^*^
Saturated fatty acid	17.56 (6.49)	18.57 (8.25)	0.004^*^
Monounsaturated fatty acid	26.10 (8.91)	27.77 (11.20)	0.001^*^
Polyunsaturated fatty acid	12.20 (5.29)	13.12 (6.55)	0.001^*^

*P value for the t-test

^†^ *P* value for the chi-square test

^‡^ Others include single, divorced, widowed, and separated

When contrasting the highest with lowest quintiles, intakes of MUFA (OR: 0.33, 95% CI: 0.21–0.51), PUFA (OR: 0.32, 95% CI: 0.20–0.51), and TFA (OR: 0.44, 95% CI: 0.28–0.70) were significantly associated with a decreased risk of ESCC after adjustment for the confounders (Table 2). A significant exposure-response trend was also found in MUFA, PUFA, and TFA (*P*_-trend_ <  0.05). Every 10 g/day increment intake of dietary fat (OR: 1.11, 95% CI: 1.04–1.19) and MUFA (OR: 0.70, 95% CI: 0.59–0.81) were related to the ESCC risk after considering the confounders.

**Table 2:** Association of dietary fat and fatty acids intake with risk of ESCC

Dietary items	N (controls/cases)	Crude OR (95% CI) *	Adjusted OR (95% CI) *^†^	Adjusted OR (95% CI) *^‡^
Dietary fat				
Quintile 1 (≤43.81 μg/day)	180/175	1.00	1.00	1.00
Quintile 2 (> 43.81 ~ ≤ 55.64 μg/day)	173/181	1.08 (0.80, 1.45)	0.97 (0.70, 1.33)	0.91 (0.65, 1.29)
Quintile 3 (> 55.64 ~ ≤ 65.04 μg/day)	196/158	0.83 (0.62, 1.11)	0.76 (0.55, 1.06)	0.71 (0.50, 1.01)
Quintile 4 (> 65.04 ~ ≤ 79.11 μg/day)	188/166	0.91 (0.68, 1.22)	0.79 (0.56, 1.11)	0.77 (0.53, 1.12)
Quintile 5 (> 79.11 μg/day)	155/199	1.32 (0.98, 1.78)	1.16 (0.80, 1.67)	1.28 (0.80, 2.05)
*P for trend*	0.2534	0.254	0.896	0.986
Every 10-unit increment		1.07 (1.03, 1.11)	1.05 (1.00, 1.11)	1.11 (1.04, 1.19)
Total fatty acid				
Quintile 1 (≤40.93 μg/day)	161/195	1.00	1.00	1.00
Quintile 2 (> 40.93 ~ ≤ 52.01 μg/day)	157/195	1.03 (0.76, 1.38)	0.90 (0.65, 1.25)	0.81 (0.58, 1.15)
Quintile 3 (> 52.01 ~ ≤ 60.91 μg/day)	195/159	0.67 (0.50, 0.90)	0.56 (0.40, 0.77)	0.48 (0.34, 0.69)
Quintile 4 (> 60.91 ~ ≤ 74.48 μg/day)	196/158	0.67 (0.49, 0.89)	0.53 (0.37, 0.74)	0.42 (0.29, 0.62)
Quintile 5 (> 74.48 μg/day)	183/172	0.78 (0.58, 1.04)	0.54 (0.38, 0.78)	0.44 (0.28, 0.70)
*P for trend*	0.0053	0.005	0	0
Every 10-unit increment		1.00 (0.96, 1.04)	0.95 (0.91, 1.00)	0.93 (0.87, 1.00)
Saturated fatty acid				
Quintile 1 (≤11.99 μg/day)	177/178	1.00	1.00	1.00
Quintile 2 (> 11.99 ~ ≤ 15.94 μg/day)	175/179	1.02 (0.76, 1.37)	0.94 (0.68, 1.29)	0.88 (0.62, 1.23)
Quintile 3 (> 15.94 ~ ≤ 18.6 μg/day)	187/167	0.89 (0.66, 1.19)	0.84 (0.61, 1.17)	0.76 (0.53, 1.08)
Quintile 4 (> 18.6 ~ ≤ 22.92 μg/day)	189/165	0.87 (0.65, 1.17)	0.70 (0.50, 0.98)	0.64 (0.44, 0.92)
Quintile 5 (> 22.92 μg/day)	164/190	1.15 (0.86, 1.55)	0.97 (0.67, 1.39)	0.93 (0.59, 1.47)
*P for trend*	0.7119	0.712	0.348	0.161
Every 10-unit increment		1.20 (1.06, 1.37)	1.14 (0.97, 1.34)	1.21 (0.96, 1.51)
Monounsaturated fatty acids				
Quintile 1 (≤19.48 μg/day)	158/198	1.00	1.00	1.00
Quintile 2 (> 19.48 ~ ≤ 25 μg/day)	144/209	1.16 (0.86, 1.56)	1.05 (0.76, 1.45)	0.94 (0.66, 1.33)
Quintile 3 (> 25 ~ ≤ 29.25 μg/day)	192/161	0.67 (0.50, 0.90)	0.53 (0.38, 0.73)	0.43 (0.30, 0.62)
Quintile 4 (> 29.25 ~ ≤ 35.25 μg/day)	208/147	0.56 (0.42, 0.76)	0.43 (0.31, 0.61)	0.33 (0.22, 0.47)
Quintile 5 (> 35.25 μg/day)	190/164	0.69 (0.51, 0.93)	0.45 (0.31, 0.65)	0.33 (0.21, 0.51)
*P for trend*		< 0.001	< 0.001	< 0.001
Every 10-unit increment		0.92 (0.84, 1.00)	0.81 (0.72, 0.91)	0.70 (0.59, 0.81)
Polyunsaturated fatty acid				
Quintile 1 (≤8.88 μg/day)	148/207	1.00	1.00	1.00
Quintile 2 (> 8.88 ~ ≤ 10.85 μg/day)	166/188	0.81 (0.60, 1.09)	0.73 (0.53, 1.01)	0.66 (0.47, 0.93)
Quintile 3 (> 10.85 ~ ≤ 13.22 μg/day)	205/148	0.52 (0.38, 0.70)	0.41 (0.29, 0.57)	0.36 (0.24, 0.51)
Quintile 4 (> 13.22 ~ ≤ 16.55 μg/day)	178/176	0.71 (0.53, 0.95)	0.54 (0.38, 0.77)	0.49 (0.33, 0.73)
Quintile 5 (> 16.55 μg/day)	195/160	0.59 (0.44, 0.79)	0.38 (0.26, 0.56)	0.32 (0.20, 0.51)
*P for trend*		< 0.001	< 0.001	< 0.001
Every 10-unit increment		0.93 (0.80, 1.08)	0.80 (0.66, 0.96)	0.85 (0.66, 1.10)

*Unconditional logistic regression was used to estimate odds ratio (OR) and 95% confidence interval (95% CI)

^†^Adjustment for age, gender, body mass index (kg/m^2^), family cancer history, cigarette smoking, alcohol drinking, education, marital status, and total calorie intake (MJ)

^‡^ Adjustment for age, gender, body mass index (kg/m^2^), family cancer history, cigarette smoking, alcohol drinking, education, marital status, total calorie intake (MJ), prudent pattern score, and healthy pattern score

Similar findings were demonstrated when the energy-adjusted intake of dietary fat and fatty acids were used in place of unadjusted intake (Table 3). The stratified analysis by cigarette smoking showed a consistent association of interest between the subgroups (Table 4). When stratified by alcohol drinking (Table 5), a significantly reduced odds of ESCC were observed among those with the highest quintile intake of TFA, MUFA, and PUFA in both non-drinkers and drinkers; however, when contrasting the highest with lowest quintiles, SFA was associated with a decreased risk of ESCC (OR: 0.39, 95% CI: 0.19–0.81, *P*_*-trend*_ = 0.002) among non-drinkers but not among drinkers. Similarly, every 10-unit increment of SFA intake was related to a reduced ESCC risk among non-drinkers but not among drinkers. The possible multiplicative interactions between alcohol drinking, cigarette smoking, and intake of dietary fat and fatty acid were examined by using the likelihood ratio test, respectively; however, no interplay was displayed (all *P*_*-interaction*_ > 0.05).

**Table 3:** Association of energy-adjusted intake of dietary fat and fatty acids with the risk of ESCC

Dietary items^§^	N (controls/cases)	Crude OR (95% CI) *	Adjusted OR (95% CI) *^†^	Adjusted OR (95% CI) *^‡^
Dietary fat				
Quintile 1 (≤48.25)	179/192	1.00	1.00	1.00
Quintile 2 (> 48.25 ~ ≤ 57.18)	178/148	0.78 (0.57, 1.04)	0.72 (0.52, 1.00)	0.79 (0.55, 1.11)
Quintile 3 (> 57.18 ~ ≤ 65.89)	178/176	0.92 (0.69, 1.23)	0.98 (0.72, 1.35)	1.07 (0.76, 1.50)
Quintile 4 (> 65.89 ~ ≤ 75.94)	178/142	0.74 (0.55, 1.00)	0.74 (0.54, 1.03)	0.78 (0.54, 1.11)
Quintile 5 (> 75.94)	179/221	1.15 (0.87, 1.53)	1.10 (0.81, 1.49)	1.22 (0.83, 1.80)
*P for trend*		0.391	0.486	0.426
Every 10-unit increment		1.06 (1.01, 1.11)	1.05 (1.00, 1.11)	1.11 (1.04, 1.19)
Total fatty acid				
Quintile 1 (≤46.05)	179/227	1.00	1.00	1.00
Quintile 2 (> 46.05 ~ ≤ 55.14)	178/184	0.82 (0.61, 1.08)	0.77 (0.56, 1.04)	0.78 (0.56, 1.08)
Quintile 3 (> 55.14 ~ ≤ 63.65)	178/176	0.78 (0.59, 1.04)	0.80 (0.59, 1.10)	0.77 (0.55, 1.08)
Quintile 4 (> 63.65 ~ ≤ 73.51)	178/128	0.57 (0.42, 0.76)	0.58 (0.42, 0.80)	0.51 (0.35, 0.73)
Quintile 5 (> 73.51)	179/164	0.72 (0.54, 0.96)	0.67 (0.49, 0.91)	0.57 (0.38, 0.85)
*P for trend*		0.002	0.003	0.001
Every 10-unit increment		0.96 (0.92, 1.01)	0.95 (0.91, 1.00)	0.93 (0.87, 1.00)
Saturated fatty acid				
Quintile 1 (≤13.25)	179/190	1.00	1.00	1.00
Quintile 2 (> 13.25 ~ ≤ 16.58)	178/173	0.92 (0.68, 1.23)	0.88 (0.64, 1.21)	0.92 (0.65, 1.29)
Quintile 3 (> 16.58 ~ ≤ 19.04)	178/167	0.88 (0.66, 1.19)	0.88 (0.64, 1.22)	0.92 (0.65, 1.30)
Quintile 4 (> 19.04 ~ ≤ 21.95)	178/140	0.74 (0.55, 1.00)	0.77 (0.56, 1.07)	0.81 (0.56, 1.16)
Quintile 5 (> 21.95)	179/209	1.10 (0.83, 1.46)	1.02 (0.75, 1.39)	0.96 (0.65, 1.42)
*P for trend*		0.934	0.905	0.601
Every 10-unit increment		1.17 (1.00, 1.36)	1.14 (0.97, 1.34)	1.21 (0.96, 1.51)
Monounsaturated fatty acid				
Quintile 1 (≤22.03)	179/236	1.00	1.00	1.00
Quintile 2 (> 22.03 ~ ≤ 26.8)	178/226	0.96 (0.73, 1.27)	0.98 (0.72, 1.32)	0.93 (0.67, 1.29)
Quintile 3 (> 26.8 ~ ≤ 30.69)	178/154	0.66 (0.49, 0.88)	0.65 (0.47, 0.89)	0.58 (0.41, 0.81)
Quintile 4 (> 30.69 ~ ≤ 35.23)	178/121	0.52 (0.38, 0.70)	0.54 (0.39, 0.75)	0.45 (0.31, 0.64)
Quintile 5 (> 35.23)	179/142	0.60 (0.45, 0.81)	0.56 (0.41, 0.77)	0.41 (0.27, 0.62)
*P for trend*		< 0.001	< 0.001	< 0.001
Every 10-unit increment		0.83 (0.74, 0.92)	0.81 (0.72, 0.91)	0.70 (0.59, 0.81)
Polyunsaturated fatty acid				
Quintile 1 (≤10.11)	179/260	1.00	1.00	1.00
Quintile 2 (> 10.11 ~ ≤ 11.77)	178/163	0.63 (0.47, 0.84)	0.66 (0.48, 0.91)	0.68 (0.48, 0.94)
Quintile 3 (> 11.77 ~ ≤ 13.76)	178/151	0.58 (0.44, 0.78)	0.62 (0.45, 0.85)	0.62 (0.44, 0.89)
Quintile 4 (> 13.76 ~ ≤ 16.42)	178/164	0.63 (0.48, 0.84)	0.69 (0.5, 0.94)	0.64 (0.45, 0.91)
Quintile 5 (> 16.42)	179/141	0.54 (0.40, 0.73)	0.57 (0.41, 0.78)	0.56 (0.37, 0.83)
*P for trend*		< 0.001	0.001	0.007
Every 10-unit increment		0.80 (0.67, 0.95)	0.80 (0.66, 0.96)	0.85 (0.66, 1.10)

*Unconditional logistic regression was used to estimate odds ratio (OR) and 95% confidence interval (95%CI)

^†^Adjustment for age, gender, body mass index (kg/m^2^), family cancer history, cigarette smoking, alcohol drinking, education, marital status, and total calorie intake (MJ)

^‡^ Adjustment for age, gender, body mass index (kg/m^2^), family cancer history, cigarette smoking, alcohol drinking, education, marital status, total calorie intake (MJ), prudent pattern score, and healthy pattern score

^§^ Residual models, the energy-adjusted dietary intake was equal to the residual of the regression of a food component intake on calorie intake plus the mean of this food specific-component

**Table 4:** Stratified analysis by cigarette smoking on the association of dietary fat and fatty acids intake with the risk of ESCC

	Non-smoker			Smoker		
	N (controls/cases)	Crude OR (95% CI) ^*^	Adjusted OR (95% CI) ^*†^	N (controls/cases)	Crude OR (95% CI) ^*^	Adjusted OR (95% CI) ^*†^
Dietary fat						
Quintile 1 (≤43.81 μg/day)	123/80	1.00	1.00	57/95	1.00	1.00
Quintile 2 (> 43.81 ~ ≤ 55.64 μg/day)	94/71	1.16 (0.76, 1.76)	1.04 (0.64, 1.67)	79/110	0.84 (0.54, 1.29)	0.72 (0.43, 1.20)
Quintile 3 (> 55.64 ~ ≤ 65.04 μg/day)	108/58	0.83 (0.54, 1.26)	0.78 (0.47, 1.29)	88/100	0.68 (0.44, 1.05)	0.59 (0.35, 1.00)
Quintile 4 (> 65.04 ~ ≤ 79.11 μg/day)	118/65	0.85 (0.56, 1.28)	0.75 (0.44, 1.28)	70/101	0.87 (0.55, 1.35)	0.72 (0.41, 1.27)
Quintile 5 (> 79.11 μg/day)	91/69	1.17 (0.76, 1.78)	1.02 (0.52, 1.99)	64/130	1.22 (0.78, 1.90)	1.41 (0.71, 2.82)
*P for trend*		0.946	0.472		0.294	0.638
Every 10-unit increment		1.05 (1.00, 1.11)	1.10 (1.00, 1.21)		1.06 (1.00, 1.12)	1.11 (1.00, 1.23)
Total fatty acid						
Quintile 1 (≤40.93 μg/day)	108/87	1.00	1.00	53/108	1.00	1.00
Quintile 2 (> 40.93 ~ ≤ 52.01 μg/day)	91/76	1.04 (0.68, 1.57)	0.86 (0.53, 1.38)	66/119	0.88 (0.57, 1.38)	0.68 (0.40, 1.13)
Quintile 3 (> 52.01 ~ ≤ 60.91 μg/day)	107/61	0.71 (0.46, 1.08)	0.53 (0.32, 0.87)	88/98	0.55 (0.35, 0.84)	0.40 (0.24, 0.68)
Quintile 4 (> 60.91 ~ ≤ 74.48 μg/day)	121/59	0.61 (0.40, 0.92)	0.41 (0.23, 0.70)	75/99	0.65 (0.41, 1.01)	0.39 (0.22, 0.68)
Quintile 5 (> 74.48 μg/day)	107/60	0.70 (0.45, 1.06)	0.35 (0.18, 0.69)	76/112	0.72 (0.46, 1.12)	0.46 (0.23, 0.90)
*P for trend*		0.009	< 0.001		0.066	0.002
Every 10-unit increment		0.99 (0.93, 1.05)	0.92 (0.83, 1.02)		0.99 (0.93, 1.05)	0.92 (0.83, 1.02)
Saturated fatty acid						
Quintile 1 (≤11.99 μg/day)	117/75	1.00	1.00	60/103	1.00	1.00
Quintile 2 (> 11.99 ~ ≤ 15.94 μg/day)	95/79	1.30 (0.86, 1.97)	1.13 (0.70, 1.82)	80/100	0.73 (0.47, 1.12)	0.61 (0.37, 1.01)
Quintile 3 (> 15.94 ~ ≤ 18.6 μg/day)	116/64	0.86 (0.56, 1.31)	0.79 (0.48, 1.29)	71/103	0.85 (0.54, 1.31)	0.66 (0.39, 1.11)
Quintile 4 (> 18.6 ~ ≤ 22.92 μg/day)	111/62	0.87 (0.57, 1.33)	0.66 (0.39, 1.11)	78/103	0.77 (0.50, 1.19)	0.55 (0.32, 0.94)
Quintile 5 (> 22.92 μg/day)	95/63	1.03 (0.67, 1.59)	0.70 (0.35, 1.38)	69/127	1.07 (0.69, 1.65)	1.02 (0.53, 1.97)
*P for trend*		0.491	0.068		0.602	0.673
Every 10-unit increment		1.15 (0.95, 1.38)	1.13 (0.81, 1.57)		1.18 (0.98, 1.42)	1.23 (0.89, 1.70)
Monounsaturated fatty acids						
Quintile 1 (≤19.48 μg/day)	107/89	1.00	1.00	51/109	1.00	1.00
Quintile 2 (> 19.48 ~ ≤ 25 μg/day)	83/84	1.22 (0.80, 1.84)	1.04 (0.65, 1.68)	61/125	0.96 (0.61, 1.51)	0.73 (0.44, 1.23)
Quintile 3 (> 25 ~ ≤ 29.25 μg/day)	107/58	0.65 (0.42, 1.00)	0.45 (0.27, 0.75)	85/103	0.57 (0.36, 0.88)	0.38 (0.22, 0.64)
Quintile 4 (> 29.25 ~ ≤ 35.25 μg/day)	123/57	0.56 (0.36, 0.85)	0.32 (0.19, 0.55)	85/90	0.50 (0.32, 0.77)	0.29 (0.16, 0.50)
Quintile 5 (> 35.25 μg/day)	114/55	0.58 (0.38, 0.89)	0.23 (0.12, 0.46)	76/109	0.67 (0.43, 1.04)	0.36 (0.18, 0.70)
*P for trend*		< 0.001	< 0.001		0.004	< 0.001
Every 10-unit increment		0.89 (0.78, 1.02)	0.67 (0.53, 0.84)		0.9 (0.79, 1.03)	0.68 (0.54, 0.85)
Polyunsaturated fatty acid						
Quintile 1 (≤8.88 μg/day)	98/91	1.00	1.00	50/116	1.00	1.00
Quintile 2 (> 8.88 ~ ≤ 10.85 μg/day)	104/67	0.69 (0.46, 1.05)	0.57 (0.35, 0.93)	62/121	0.84 (0.53, 1.32)	0.68 (0.41, 1.14)
Quintile 3 (> 10.85 ~ ≤ 13.22 μg/day)	106/54	0.55 (0.35, 0.84)	0.48 (0.28, 0.80)	99/94	0.41 (0.26, 0.63)	0.25 (0.14, 0.43)
Quintile 4 (> 13.22 ~ ≤ 16.55 μg/day)	111/74	0.72 (0.48, 1.08)	0.53 (0.31, 0.91)	67/102	0.66 (0.42, 1.03)	0.41 (0.23, 0.74)
Quintile 5 (> 16.55 μg/day)	115/57	0.53 (0.35, 0.82)	0.31 (0.16, 0.60)	80/103	0.55 (0.36, 0.86)	0.27 (0.13, 0.53)
*P for trend*		0.010	0.002		0.006	< 0.001
Every 10-unit increment		0.95 (0.76, 1.17)	0.92 (0.64, 1.30)		0.87 (0.70, 1.09)	0.72 (0.49, 1.05)

*Unconditional logistic regression was used to estimate the odds ratio (OR) and 95% confidence interval (95% CI)

^†^Adjustment for age, gender, body mass index (kg/m^2^), family cancer history, alcohol drinking, education, marital status, prudent pattern score, healthy pattern score, and total calorie intake (MJ)

**Table 5:** Stratified analysis by alcohol drinking on the association of dietary fat and fatty acids intake with the risk of ESCC

	Non-drinker			Drinker		
	N (controls/cases)	Crude OR (95% CI) *	Adjusted OR (95% CI) *^†^	N (controls/cases)	Crude OR (95% CI) *	Adjusted OR (95% CI) *^†^
Dietary fat						
Quintile 1 (≤43.81 μg/day)	121/78	1.00	1.00	59/97	1.00	1.00
Quintile 2 (> 43.81 ~ ≤ 55.64 μg/day)	81/68	1.30 (0.85, 2.00)	1.16 (0.72, 1.87)	92/113	0.75 (0.49, 1.14)	0.69 (0.42, 1.14)
Quintile 3 (> 55.64 ~ ≤ 65.04 μg/day)	100/55	0.85 (0.55, 1.32)	0.78 (0.47, 1.30)	96/103	0.65 (0.42, 1.00)	0.60 (0.36, 1.00)
Quintile 4 (> 65.04 ~ ≤ 79.11 μg/day)	96/60	0.97 (0.63, 1.49)	0.69 (0.40, 1.19)	92/106	0.70 (0.46, 1.07)	0.62 (0.35, 1.07)
Quintile 5 (> 79.11 μg/day)	78/55	1.09 (0.70, 1.71)	0.57 (0.27, 1.18)	77/144	1.14 (0.74, 1.74)	1.69 (0.86, 3.33)
*P for trend*		0.834	0.053		0.45	0.41
Every 10-unit increment		1.04 (0.98, 1.10)	0.91 (0.81, 1.02)		1.06 (1.01, 1.12)	1.19 (1.08, 1.31)
Total fatty acid						
Quintile 1 (≤40.93 μg/day)	111/86	1.00	1.00	50/109	1.00	1.00
Quintile 2 (> 40.93 ~ ≤ 52.01 μg/day)	73/72	1.27 (0.83, 1.96)	0.96 (0.59, 1.56)	84/123	0.67 (0.43, 1.03)	0.59 (0.35, 0.98)
Quintile 3 (> 52.01 ~ ≤ 60.91 μg/day)	101/54	0.69 (0.45, 1.06)	0.50 (0.30, 0.83)	94/105	0.51 (0.33, 0.79)	0.39 (0.23, 0.65)
Quintile 4 (> 60.91 ~ ≤ 74.48 μg/day)	101/57	0.73 (0.47, 1.12)	0.35 (0.20, 0.61)	95/101	0.49 (0.31, 0.75)	0.31 (0.17, 0.54)
Quintile 5 (> 74.48 μg/day)	90/47	0.67 (0.43, 1.06)	0.16 (0.07, 0.35)	93/125	0.62 (0.40, 0.94)	0.49 (0.25, 0.94)
*P for trend*	0.0108	0.011	< 0.001		0.017	0.004
Every 10-unit increment		0.98 (0.92, 1.04)	0.71 (0.62, 0.81)		0.99 (0.94, 1.05)	0.98 (0.89, 1.09)
Saturated fatty acid						
Quintile 1 (≤11.99 μg/day)	120/79	1.00	1.00	57/99	1.00	1.00
Quintile 2 (> 11.99 ~ ≤ 15.94 μg/day)	77/72	1.42 (0.93, 2.18)	1.17 (0.73, 1.88)	98/107	0.63 (0.41, 0.96)	0.60 (0.36, 0.99)
Quintile 3 (> 15.94 ~ ≤ 18.6 μg/day)	99/57	0.87 (0.57, 1.35)	0.75 (0.45, 1.24)	88/110	0.72 (0.47, 1.10)	0.63 (0.37, 1.06)
Quintile 4 (> 18.6 ~ ≤ 22.92 μg/day)	101/57	0.86 (0.56, 1.32)	0.53 (0.31, 0.89)	88/108	0.71 (0.46, 1.09)	0.54 (0.31, 0.93)
Quintile 5 (> 22.92 μg/day)	79/51	0.98 (0.62, 1.54)	0.39 (0.19, 0.81)	85/139	0.94 (0.62, 1.44)	1.08 (0.56, 2.08)
*P for trend*		0.319	0.002		0.712	0.961
Every 10-unit increment		1.10 (0.91, 1.33)	0.56 (0.37, 0.83)		1.19 (1.00, 1.42)	1.07 (0.96, 2.01)
Monounsaturated fatty acids						
Quintile 1 (≤19.48 μg/day)	107/90	1.00	1.00	51/108	1.00	1.00
Quintile 2 (> 19.48 ~ ≤ 25 μg/day)	72/77	1.27 (0.83, 1.95)	0.88 (0.54, 1.43)	72/132	0.87 (0.56, 1.34)	0.77 (0.46, 1.29)
Quintile 3 (> 25 ~ ≤ 29.25 μg/day)	93/53	0.68 (0.44, 1.05)	0.43 (0.26, 0.73)	99/108	0.52 (0.33, 0.79)	0.37 (0.22, 0.62)
Quintile 4 (> 29.25 ~ ≤ 35.25 μg/day)	109/54	0.59 (0.38, 0.90)	0.24 (0.14, 0.42)	99/93	0.44 (0.29, 0.68)	0.26 (0.15, 0.46)
Quintile 5 (> 35.25 μg/day)	95/42	0.53 (0.33, 0.83)	0.09 (0.04, 0.19)	95/122	0.61 (0.39, 0.93)	0.43 (0.23, 0.82)
*P for trend*		< 0.001	< 0.001		0.001	< 0.001
Every 10-unit increment		0.87 (0.76, 1.00)	0.37 (0.27, 0.49)		0.9 (0.8, 1.02)	0.78 (0.63, 0.97)
Polyunsaturated fatty acid						
Quintile 1 (≤8.88 μg/day)	97/84	1.00	1.00	51/123	1.00	1.00
Quintile 2 (> 8.88 ~ ≤ 10.85 μg/day)	92/70	0.88 (0.57, 1.35)	0.67 (0.41, 1.08)	74/118	0.66 (0.43, 1.02)	0.55 (0.33, 0.92)
Quintile 3 (> 10.85 ~ ≤ 13.22 μg/day)	102/51	0.58 (0.37, 0.90)	0.42 (0.24, 0.72)	103/97	0.39 (0.25, 0.60)	0.25 (0.15, 0.43)
Quintile 4 (> 13.22 ~ ≤ 16.55 μg/day)	88/65	0.85 (0.55, 1.32)	0.43 (0.24, 0.77)	90/111	0.51 (0.33, 0.78)	0.35 (0.20, 0.61)
Quintile 5 (> 16.55 μg/day)	97/46	0.55 (0.35, 0.86)	0.15 (0.07, 0.32)	98/114	0.48 (0.31, 0.73)	0.30 (0.16, 0.58)
*P for trend*		0.017	< 0.001		0.001	< 0.001
Every 10-unit increment		0.90 (0.71, 1.13)	0.48 (0.30, 0.75)		0.89 (0.72, 1.09)	0.96 (0.67, 1.36)

*Unconditional logistic regression was used to estimate odds ratio (OR) and 95% confidence interval (95%CI)

^†^Adjustment for age, gender, body mass index (kg/m^2^), family cancer history, cigarette smoking, education, marital status, prudent pattern score, healthy pattern score, and total calorie intake (MJ)

## Discussion

This study revealed that higher dietary intake of TFA, MUFA, and PUFA was related to a lower risk of ESCC, with a noticeable exposure-response trend. Whereas, the protective effect of SFA on ESCC risk was only observed among non-drinkers.

Growing evidence highlights the effect of MUFA and PUFA on cancer prevention from both animal and epidemiological studies, which was mainly due to the roles by the saponifiable components [8, 24–28]. In line with epidemiological studies in European and American countries [26–28], our study found a negative association between MUFA or PUFA intake and ESCC risk, though food sources of MUFA and PUFA and dietary patterns between Chinese and Euro-Americans differed remarkably [29, 30]. Sensitivity analysis also yielded similar results when using energy-adjusted intake as exposure, indicating the robustness of the results. A meta-analysis combining 10 studies [31] revealed that olive oil consumption, rich in MUFA, brought forward lower odds of developing cancer of all type, and thus indicated the potential anticancer effect of using MUFA and PUFA in preventing the occurrence of ESCC. The possible anti-cancer mechanism might be due to that MUFA and PUFA can promote apoptosis of cancer cells and maintain metabolisms of normal cells by triggering both endogenous and exogenous apoptosis pathways [32, 33]. Besides, it was found that PUFA contained multiple unsaturated double bonds, which were vulnerable to the active oxygen radicals and subsequently led to non-enzymatic lipid peroxidation reaction to produce a variety of high active acetaldehyde intermediates [34, 35]. These molecules can be quickly linked to the protein of the cell membrane to form lipid peroxidation end products, damaging the stability and function of cancer cell membrane protein [34–36], which in turn may exert an antineoplastic effect.

When all subjects were considered, no significant association was found between SFA intake and risk of ESCC. This finding was consistent with the research results from other cohort or case-control studies [26, 37]. The sensitivity analysis by using energy-adjusted intake and stratified analysis by smoking yield similar results, indicating the robustness of the results. However, the stratified analysis found that among non-drinkers but not among drinkers, a higher-level intake of dietary SFA made a positive impact on a reduced risk of ESCC. This might be attributed to the fact that alcohol intake was evidenced to significantly increase the incidence of ESCC [38] and studies have found that dietary saturated fatty acids played an important role in reversing inflammatory changes and altering some deleterious effects of ethanol [39, 40]. It was also reported that a diet with abundant SFA could effectively reverse alcohol-induced necrosis, inflammation, and fibrosis [39]. However, this should be confirmed by longitudinal studies. This study found that TFA was related to a reduced risk of ESCC, however, owing to the complex composition of the components and the differences in the functions of each component, more investigations are needed to verify the results.

The latest systematic review and meta-analysis with observational studies also found that the intake of dietary fat had nothing to do with ESCC risk [9]. Similarly, results from many epidemiological studies did not meet with the hypothesis that the lower total fat intake, the smaller occurrence of suffering from cancer [30]. In this present study, it was observed that every 10-unit of dietary fat was associated with 11% increased risk; however, the exposure-response trend was not significant. Probably, only those with a very high dietary fat intake have an elevated risk of ESCC. Thus, the relation between dietary fat and ESCC risk needs to be further evaluated.

### Comparisons with other studies and what does the current work add to the existing knowledge

As far as we are concerned, this is the first study in Eastern Asia that explores the associations of dietary fat and fatty acids intake with ESCC risk. Consistent with many western studies, this current study added up the evidence that dietary consumption of PUFA and MUFA may have a protective effect on ESCC in the Chinese population. What’s more, this study provides new evidence that higher levels of dietary SFA intake laid an impact on a reduced risk of ESCC among non-drinkers in a high-risk area.

### Strengths and limitations

The strengths of this study are listed in the following statement. Firstly, the cases included in this study accounted for about 70% of all ESCC patients during the recruited period; there was no remarkable difference in age and gender between patients included and not included. The controls were randomly selected through multistage sampling from the same communities with cases. All controls received an endoscopic examination within the past month and no esophageal cancer or precancerous lesions was observed. These could help to reduce the selection bias and to ensure the representativeness and comparability of the study objects. Secondly, incident cases were recruited and the interview was performed within seven days after the cases were diagnosed as ESCC, which can help to lower recall bias. Thirdly, confounders were collected as many as possible and multivariate analysis was used, which can control the potential confounding effects brought by these factors on the relationship of interest. Fourthly, the study population lived in remote rural areas, so their living environment was less polluted by modern industrial activities, and their dietary habits did not change significantly in the last decades. These provided an ideal research site for investigating the relationship between diet and esophageal cancer. Last but not the least, the investigation was conducted by a face-to-face interview and by trained interviewers with local dialect, the questionnaire was successfully collected from each participant, and missing data rarely appeared.

There are also some limitations. Like other case-control studies, information bias especially recall biacontrols being interviewed was less than may be a concern. It was a challenge to collect accurate dietary information five years before the interview for controls or before the diagnosis for ESCC cases. Several measures were adopted to minimize the information bias. The study was introduced as a nutrition and health survey rather than a cancer study, and this approach led the study subjects and interviewers to be blinded to the hypothesis and the research objective. Food pictures and bowls with scales were provided to help the subjects recall and estimate the amount of each food consumed. The interviewers were trained strictly to collect data from the cases and the controls with the same approach, and the time interval between the cases and controls being interviewed was less than one month. The dose-response relationship between diet fat or fatty acids intake and ESCC risk suggested that the collected dietary exposure was likely to be genius. The serum indicators of dietary and fatty acids were not collected, and the measurement of dietary factors only relied on FFQ, which may have posed another limitation; nevertheless, other studies have validated that dietary fat composition obtained from FFQ could reasonably reflect plasma fatty acid composition [41, 42].

## Conclusion

The results suggested that high levels of MUFA and PUFA intake were related to a lower risk of ESCC, whereas the protective effect of SFA was only observed among non-drinkers. This study indicated that strategic nutritional programs should consider food rich in unsaturated fatty acids to mitigate the occurrence of ESCC. Large-scale prospective studies, including cohort studies and intervention studies, are needed to keep confirming the roles of fatty acid intake and their related metabolic biomarkers in the occurrence of ESCC.

## Supplementary Information

**Additional file 1: Supplementary Table S1.** Factor loadings for the relationship between food groups and factors representing dietary patterns*. *Principal component and factor analysis was performed based on 21 dietary items. With orthogonal rotation, the factor loading scores are identical to the correlation coefficients. The magnitude of each loading indicates the importance of the corresponding items to the factor. Loadings ≥0.42 were shown in bold typeface. Prudent pattern and healthy pattern were defined and the pattern scores were calculated by using weighted methods. Pattern Score = $\sum \limits_1^{21}{variable}_i\times {weight}_i$; *variable* represents each food item intake; *weight* means the factor loading.

## Data Availability

The data that support the findings of this study are available on request from the corresponding author.

## Abbreviations

EC: Esophageal cancer
ESCC: Esophageal squamous cell carcinoma
SFA: Saturated fatty acid
MUFA: Monounsaturated fatty acid
PUFA: Polyunsaturated fatty acid
TFA: Total fatty acid
OR: Odds ratio
CI: Confidence interval
FFQ: Food frequency questionnaire
BMI: Body mass index
