# Supplementary material for: Dietary intake of monounsaturated and polyunsaturated fatty acids is related to the reduced risk of esophageal squamous cell carcinoma
Source: Lipids Health Dis. 2022 Feb 27;21:25. doi: 10.1186/s12944-022-01624-y (PMC8883658; doi:10.1186/s12944-022-01624-y)
Supplement: Supplementary file 1 — Additional file 1: Supplementary Table S1. Factor loadings for the relationship between food groups and factors representing dietary patterns*. *Principal component and factor analysis was performed based on 21 dietary items. With orthogonal rotation, the factor loading scores are identical to the correlation coefficients. The magnitude of each loading indicates the importance of the corresponding items to the factor. Loadings ≥0.42 were shown in bold typeface. Prudent pattern and healthy pattern were defined and the pattern scores were calculated by using weighted methods. Pattern Score = $\sum \limits_1^{21}{variable}_i\times {weight}_i$∑121variablei×weighti; variable represents each food item intake; weight means the factor loading. [file 12944_2022_1624_MOESM1_ESM.docx]

**Supplementary Materials**

Supplementary Table S1. Factor loadings for the relationship between food groups and factors representing dietary patterns*

|  | Prudent pattern | Healthy pattern | communality | Uniquenesses | complexity |
| --- | --- | --- | --- | --- | --- |
| Rice | -0.01 | 0.07 | 0.01 | 0.99 | 1.03 |
| Wheat | 0.08 | -0.03 | 0.01 | 0.99 | 1.27 |
| Corn | 0.11 | -0.01 | 0.01 | 0.99 | 1.03 |
| Crops | 0.11 | 0.02 | 0.01 | 0.99 | 1.05 |
| Preserved vegetables | 0.32 | -0.43 | 0.29 | 0.71 | 1.83 |
| Pickled vegetables | 0.29 | 0.05 | 0.09 | 0.91 | 1.06 |
| Salted eggs | 0.42 | 0.02 | 0.18 | 0.82 | 1.00 |
| Salted meat | 0.45 | -0.15 | 0.23 | 0.77 | 1.23 |
| Fruits | 0.15 | 0.64 | 0.43 | 0.57 | 1.11 |
| Fresh vegetables | 0.11 | 0.45 | 0.22 | 0.78 | 1.12 |
| Red meat | 0.52 | 0.24 | 0.33 | 0.67 | 1.43 |
| White meat | 0.71 | -0.09 | 0.51 | 0.49 | 1.03 |
| Fish and shrimp | 0.82 | 0.07 | 0.68 | 0.32 | 1.01 |
| Fresh eggs | 0.33 | 0.35 | 0.24 | 0.76 | 1.99 |
| Tofu | 0.49 | 0.34 | 0.35 | 0.65 | 1.80 |
| Dred beans | 0.40 | 0.43 | 0.34 | 0.66 | 1.99 |
| Seeds and nuts | 0.34 | 0.32 | 0.22 | 0.78 | 1.99 |
| Tea | 0.25 | -0.32 | 0.17 | 0.83 | 1.87 |
| Beer | 0.25 | -0.19 | 0.10 | 0.90 | 1.89 |
| White liquor | 0.31 | -0.39 | 0.25 | 0.75 | 1.90 |
| Edible oil | 0.00 | -0.50 | 0.25 | 0.75 | 1.00 |
| SS loadings | 2.92 | 1.98 |  |  |  |
| Proportion Explained (%) | 59.58 | 40.42 |  |  |  |
| Cumulative Proportion (%) | 59.58 | 100.00 |  |  |  |

*Principal component and factor analysis was performed based on 21 dietary items. With orthogonal rotation, the factor loading scores are identical to the correlation coefficients. The magnitude of each loading indicates the importance of the corresponding items to the factor. Loadings ≥ 0.42 were shown in bold typeface. Prudent pattern and healthy pattern were defined and the pattern scores were calculated by using weighted methods.

Pattern Score = $\sum_{1}^{21} {variable}_{i}\times{weight}_{i}$; ***variable*** represents each food item intake; ***weight*** means the factor loading.
